# Improved Detection of Tryptic Peptides from Tissue Sections Using Desorption Electrospray Ionization Mass Spectrometry Imaging

**DOI:** 10.1021/jasms.4c00006

**Published:** 2024-04-11

**Authors:** Heather Bottomley, Jonathan Phillips, Philippa Hart

**Affiliations:** † Living Systems Institute, Department of Biosciences, University of Exeter, Stocker Road, Exeter EX4 4QD, U.K.; ‡ Medicines Discovery Catapult, Alderley Park, Block 35, Mereside, Macclesfield SK10 4ZF, U.K.

## Abstract

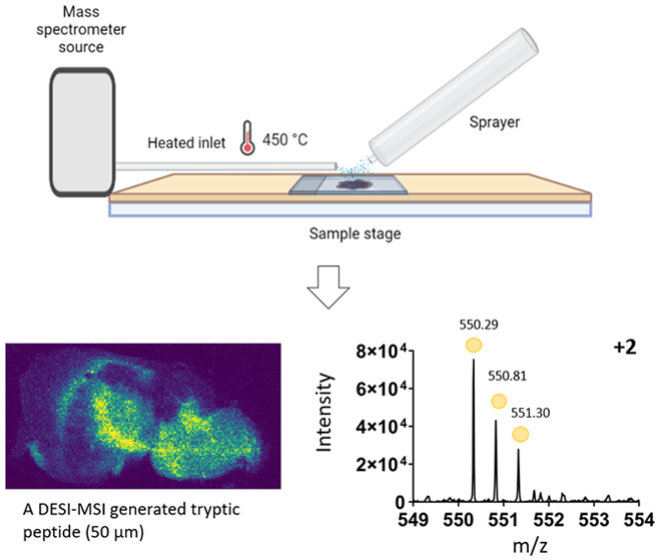

DESI-MSI is an ambient ionization technique used frequently for the detection of lipids, small molecules, and drug targets. Until recently, DESI had only limited use for the detection of proteins and peptides due to the setup and needs around deconvolution of data resulting in a small number of species being detected at lower spatial resolution. There are known differences in the ion species detected using DESI and MALDI for nonpeptide molecules, and here, we identify that this extends to proteomic species. DESI MS images were obtained for tissue sections of mouse and rat brain using a precommercial heated inlet (approximately 450 °C) to the mass spectrometer. Ion mobility separation resolved spectral overlap of peptide ions and significantly improved the detection of multiply charged species. The images acquired were of pixel size 100 μm (rat brain) and 50 μm (mouse brain), respectively. Observed tryptic peptides were filtered against proteomic target lists, generated by LC–MS, enabling tentative protein assignment for each peptide ion image. Precise localizations of peptide ions identified by DESI and MALDI were found to be comparable. Some spatially localized peptides ions were observed in DESI that were not found in the MALDI replicates, typically, multiply charged species with a low mass to charge ratio. This method demonstrates the potential of DESI-MSI to detect large numbers of tryptic peptides from tissue sections with enhanced spatial resolution when compared to previous DESI-MSI studies.

## Introduction

The ability to precisely identify and spatially locate proteins and peptides in tissue is of critical importance to fundamental biological research, e.g., drug discovery, with the identification of drug targets^1^ and in clinical medicine, e.g., discovery of diagnostic biomarkers.^2^ These molecules play a decisive role in the function of different cellular processes including maintaining the cell structure, transporting essential molecules, and signal transduction.^3^ Determining the distribution of these proteins and peptides in the tissue can be instrumental in understanding and locating their mechanism of action within the different features of tissues.^4^

Mass spectrometry imaging (MSI) is an analytical technique that can be applied to detect the molecular ions and convey their spatial distribution in tissue sections.^5^ MSI is implemented by scanning the tissue surface collecting ions and a mass spectrum at set increments.^6^ These mass spectra can then be used to create a reconstructed image by displaying the differences in intensities of the selected ions across the area acquired. The surface distance between each recorded mass spectrum while scanning will determine the pixel size of the resulting image.^7^ MSI has been used to detect endogenous molecules such as lipids,^8^ proteins,^9^ peptides,^10^ metabolites,^11^ and glycans^12^ as well as exogenous molecules like certain small molecule drugs^13^ and polymers.^14^

Desorption electrospray ionization (DESI) is an ambient ionization technique with which tissue sections can be analyzed without sample pretreatment. When coupled to a mass spectrometer (MS), DESI-MS allows atmospheric detection of ions from tissue surfaces.^15^ DESI-MSI has been routinely used to detect and spatially map lipids^16^ and small molecules^17^ after its introduction in 2004.^18^ Additionally, it has been used frequently to accurately detect drug targets with good spatial resolution.^19,20^ More recently, proteins and endogenous peptides have been successfully detected and spatially mapped using DESI-MSI in rat liver sections^21^ and mouse brain sections.^22^ Intact proteins have been detected and spatially mapped by nano-DESI in rat kidney sections^23,24^ and in rat brain sections.^24,25^

Matrix-assisted laser desorption/ionization (MALDI) is a soft-ionization technique requiring samples to be coated with a chemical matrix which will cocrystallize with analytes in the same surface.^26^ A laser beam ablates both matrix and analyte from the surface. Upon ionization, mostly singly charged species are generated then separated by *m*/*z* in the mass analyzer.^27^ MALDI-MSI is more widely used for protein and peptide detection, mainly due to its ability to detect species of larger molecular weight and also in part because the spatial resolution of the images generated is typically greater than for DESI-MSI, yielding localized images of specific peptides and proteins.^28^ The MALDI-MSI workflow has some strengths; notably it requires very little manual optimization and standard settings are in place^29^ for detecting molecules in a variety of tissue samples. Despite these advantages, MALDI-MSI can only usually detect singly charged species; therefore, multiply charged peptides may not be detected, potentially reducing the overall number of peptides that can be observed. This limited detection of multiply charged species is thought to be due to the ion clusters within the matrix that are already singly positively charged, as a result of the cluster mechanism.^30^ MALDI-MSI also has low ionization efficiencies for small molecules.^31^ Use of a DESI-MSI source stands to improve on these two limitations of MALDI-MSI by generating more multiply charged ions and by enhancing ionization of smaller peptides.^32^

Despite the use of DESI-MSI in the detection of proteins and peptides in tissues, this technique still requires more optimization to be used routinely for this. The spatial resolution of the peptides and proteins detected has not been comparable for that of lipids and small molecules (around 25 μm),^33^ as the images generated are typically not as clear or well resolved. The images of the intact proteins show better localization currently than the peptide images even though they have both been observed at 150 μm pixel resolution;^21^ however, both are much lower in resolution than those generated with MALDI-MSI which is usually around 50 μm.^34^ This has previously been due to the difficulty focusing the DESI solvent spray point.^35^ Specifically for peptides, the impact of charged droplets on to the surface is crucial for their ionization.^36^ It can be difficult to obtain good levels of sensitivity with DESI-MSI compared to MALDI-MSI due to the fine-tuning of the inlet and sprayer position, combined with the need for a heated inlet to aid peptide desolvation and ionization.^21^ Utilizing ion mobility alongside DESI-MSI is crucial for resolving multiple overlapping peaks in the mass spectrum from multiply charged species. However, employing ion mobility can sometimes result in aliasing of the Transfer T-Wave and ToF Pusher, causing artifacts and distortion in the image. Addressing this issue is imperative, especially since ion mobility is necessary for peptide identification in DESI-MSI experiments.^21^

Often in proteomic MSI, an enzymatic solution (e.g., trypsin in ammonium bicarbonate buffer) is applied to the surface of the tissu;^37,38^ this can allow for the detection of more diverse species than without digestion.^21^ A washing step is used prior to in situ digestion to remove abundant and interfering lipid signals from the tissue that can mask the typically lower peptide or protein signal.^39^ As DESI-MSI employs a charged solvent stream to desorb ions from the tissue with subsequent inlet capture; parameters involved in the setup and orientation of this capillary can affect analyte displacement in the tissue.^40^ The inlet angle is crucial to ensure sufficient capture of ions following a specific trajectory. Factors such as the sprayer angle, distance from the surface and inlet, as well as the flow and gas pressure are all importance factors. These factors must be optimized for your particular substrate or tissue on the specific instrument that you are utilizing.^21^ This can lead to a lack of peptide detection and low-quality images with delocalized ions where a suboptimal setup is employed.

High spatial resolution images of detectable peptides and proteins in the tissue are required. The appropriate resolution varies depending on the tissue and the specific question at hand. In numerous tissue-based applications, a range of 20–50 μm is generally sufficient. This is not always easily achieved in both DESI and MALDI-MSI.^21^ One major limitation for this is that the preparation steps for protein and peptide analysis, i.e., washing of tissue and tryptic digestion, often lead to delocalization of these molecules in the tissue resulting in suboptimal spatial resolution. Additionally, suppression of proteins and peptides frequently occurs due to other more abundant interfering ions in the tissue sections, limiting the number of detectable ions.^21^ Therefore, changes to the methodology are required to prevent ion suppression and delocalization. The motivation for this DESI-MSI optimization is that it requires minimal sample preparation (no matrix required) and ambient conditions, and the technique results in increased detection of both singly and multiply charged species.

Here, we present the detection of tryptic peptides at a defined spatial resolution in mouse and rat brain sections of 50 and 100 μm, respectively. Images obtained using both DESI-MSI and MALDI-MSI show highly resolved peptides that are localized to specific areas in the tissue sections. Peptides detected were then compared against a LC–MS generated proteomic target list. This allowed the peptides identified to be assigned to proteins depending on their *m*/*z*.

## Methods

### Tissue Handling and Sectioning

Mouse and rat brain tissues were obtained through studies performed at Medicines Discovery Catapult. No animals were euthanized specifically for use in this study. All applicable international, national, and/or institutional guidelines for the care and use of animals were followed. All studies were performed under Home Office Control and under compliance of the Animals (Scientific Procedures) Act 1986.

The mouse and rat brains were stored at −80 °C prior to use. Both the mouse and rat brains were then sectioned using a cryostat (Thermo Scientific, USA) at a thickness of 10 μm before successive sections were thaw mounted onto indium tin oxide (ITO) slides for MALDI imaging and SuperFrost Plus slides for DESI imaging. These sections were then left to dry for 1 h at room temperature. Unless molecules are prone to rapid degradation, such as metabolites, slides are typically kept at room temperature to allow tissues to thoroughly adhere to them, which can be advantageous for procedures involving tissue washing. Afterward, the slides were placed in a slide storage box to protect them from damage and delocalization before being returned to the −80 °C freezer until use. Sagittal sections were utilized for the mouse brain, while coronal sections were employed for the rat brain.

### Tissue Preparation

To prevent peptide and protein delocalization by condensation, the tissue slides were placed in a vacuum desiccator for 20 min following removal from the −80 °C freezer. Once the slides were dry, the interfering lipid and salt species needed to be removed from the tissue as their abundance would suppress the peptide signal. The most effective removal method was found to be Carnoy’s solution used alongside other solvent washes. Both rat and mouse brain sections were immersed for 30 s in solutions of 70% ethanol and 100% ethanol then immersed in Carnoy’s wash solution (6:3:1, ethanol:chloroform:acetic acid) for 2 min followed by 30 s of 100% ethanol, H_2_O with 0.2% trifluoroacetic acid (TFA), and 100% ethanol. This Carnoy’s wash method was compared against the ethanol rinse method with consecutive mouse brain sections. The ethanol rinse method uses 70% ethanol followed by 95% and 100%. After the subsequent ethanol washes the slides would be washed with 100% chloroform. Carnoy’s wash method was found to give a higher number of tryptic peptides when compared to the ethanol rinse method. The brain sections were then moved to the vacuum desiccator again for 4 min to remove the residual solvent wash solutions, thereby preventing delocalization of peptides.

Sequencing-grade modified trypsin (20 μg, Promega, Germany) was reconstituted in 1 mL of 50 mM ammonium bicarbonate at pH 8 with 0.1% octylglucosidase (10 μM). The reconstituted trypsin was then deposited onto the sections using a SunCollect Sprayer (SunChrom, Germany). The sprayer applied 10 trypsin layers with a nitrogen gas pressure of 2.5 bar. Once trypsin was applied, the slides were moved to a 50 °C incubator for 4 h to allow digestion of the proteins in the tissue. The amount of time and temperature used for the incubation gave the highest number of localized tryptic peptides for these experiments. Consecutive sections of mouse brain were evaluated for this conclusion, employing digestion incubation times ranging from 2 h to overnight and temperatures spanning from 37 to 50 °C.

The ITO slides for MALDI analysis required a matrix to be applied using the SunCollect sprayer. Five mg of α-Cyano-4-hydroxycinnamic acid (CHCA) (Merck, Germany) was resuspended in 50% acetonitrile, 0.1% trifluoroacetic acid (TFA), and 0.24% aniline. Aniline facilitates the amidation process at the C-terminus of peptides, thereby enhancing peptide ionization when used in conjunction with CHCA and TFA.^41^ This was then sonicated for 5 min. Twelve layers of this were sprayed with the parameters of 600 mm/min velocity, 25 mm Z height, and 2 mm line distance. There was 3 s of drying time between the following layers: 1 × 10 μL/min, 1 × 15 μL/min, and 10 × 20 μL/min.

### DESI Source Setup

Experimental data was acquired using a SYNAPT G2-Si Q-TOF mass spectrometer (Waters Corporation, UK) operated in mobility TOF mode using T-wave ion mobility separation. The mass spectrometer had a DESI source (Prosolia, USA) attached; this consisted of a moving sample stage, a spray nozzle, and a precommercial heated inlet (approximately 450 °C) to the mass spectrometer.

The solvent used in the sprayer for peptide detection was 80% acetonitrile, 19.8% H_2_O, and 0.2% formic acid. The solvent flow rate through the DESI sprayer was 1.2 μL/min with a nitrogen gas pressure of 0.5 bar and a voltage of 0.6 kV. The DESI sprayer was positioned to be approximately 4 mm away from the slide.

### SYNAPT G2-Si Instrument Settings

Mass spectra were acquired between 50 and 1,200 *m*/*z* with the SYNAPT G2-Si in positive ion sensitivity mode. The Synapt G2-Si can achieve resolving power up to 20,000 full width at half-maximum (fwhm) in sensitivity mode. Ion mobility was enabled, and these settings were applied in the trap and transfer cells. The Trap DC bias, Transfer Wave Velocity, and Transfer Wave Height were all optimized based on the *m*/*z* range to prevent aliasing of the Transfer T-Wave and ToF Pusher which can create artifacts within the data. These settings will be different depending on the instrument type used, as they are dependent on the ToF mass range. For these experiments, the *m*/*z* range was 1,200 *m*/*z*; therefore, the transfer wave velocity was set to 222 m/s to give a resulting pusher interval of 54 μs. The transfer wave height was set to 4.1 V and the trap DC bias at 45. We experimented with adjusting both the transfer wave velocity and transfer wave height, varying them around the calculated values derived from the *m*/*z*. Those listed were found to be the optimum values for preventing aliasing in the images.

The images were acquired with a pixel size of 100 μm for the rat brain sections and 50 μm for the mouse brain sections. Waters High-Definition Imaging (HDI) software (Waters Corporation, UK) was used to acquire the images. A flatbed scanner was utilized to obtain an image of the slide for acquisition set up.

### RapifleX MALDI Instrument Settings

A RapifleX MALDI-TOF mass spectrometer (Bruker, USA) with a smart beam 10 kHz laser was used to obtain MALDI spectra from consecutive sections of both the mouse and rat brains. The RapifleX MALDI has a mass resolution up to 40,000 fwhm in reflector mode. For accurate positioning of the MTP Slide Adaptor II in the Rapiflex, a flatbed scanner was used to gain a high-definition image of the slide. The RapifleX was calibrated using the Peptide Calibration Standard II (Bruker, USA). This standard was applied to the matrix and the laser power was adjusted depending on the intensity of the calibration peaks. Mass spectra were acquired in positive reflector acquisition mode between 539.70 and 3081.68 *m*/*z*. This mass spectra range aims to minimize the detection of matrix ions. In MALDI, numerous matrix ion peaks in the lower *m*/*z* range can suppress our ions of interest. Unlike in DESI, this concern does not arise, allowing us to reduce the *m*/*z* range to capture multiply charged peptide ions. The images were acquired at 100 μm for the rat brain sections and 50 μm for the mouse brain sections.

### Tissue Preparation for a Proteomic Experiment

Additional tissue was taken from the same mouse and rat brains in 10 μm sections on the cryostat (Thermo Scientific, USA). Ten of these sections were taken for the cerebellum and placed in a 2.0 mL tube, followed by the rest of the brain placed in a separate 2.0 mL tube. These tubes were stored in the −80 °C freezer until use.

The tissue in the 2.0 mL tubes was homogenized, sonicated, and centrifuged in 7 M urea lysis buffer with the resulting supernatant being collected. This was frozen overnight in acetone, and the precipitate was collected, dried, and resuspended in 7 M urea. The Pierce assay kit (Thermo Scientific, USA) was used to determine protein concentration through interpreting the absorbance readout. Ten kDa cutoff amicon filters (Merck, USA) were prewashed using 50 mM ammonium bicarbonate solution before protein loading. For reduction, samples were incubated with DTT for 45 min at 56 °C, followed by alkylation with CAA for 30 min. These samples were then incubated at room temperature in the dark. After this, the samples were washed 3 times with 50 mM ammonium bicarbonate. A 0.033 μg/μL trypsin solution was added to the samples with overnight incubation at 37 °C. The digest was stopped through the addition of formic acid to a final concentration of 0.1%. Peptide supernatants were then collected and dried down in an evaporator (Genevac, UK) and resuspended in 0.1% formic acid, for protein analysis via LC–MS/MS.

### Orbitrap Exploris Instrument Settings

Using the extracted peptide solutions, proteomic runs for both the rat and mouse brain cerebellum and the rest of the brain were conducted. The Orbitrap Exploris has a mass resolution of up to 240,000 fwhm. These experiments were performed with a 20 min water/acetonitrile LC-gradient on the Exploris 240 mass spectrometer (Thermo Scientific, USA). An 8 μL injection was used with a 0.25 mL/min flow rate on a 20 min gradient. A C18 ACQUITY UPLC HSS T3 column was used for separation with the ACQUITY UPLC I-Class PLUS System (Waters, UK). The data was collected in with a heated electrospray ionization (HESI) source in positive mode with a data-dependent acquisition.

### Post Imaging Analysis

Following MALDI analysis of the tissue sections, the matrix was removed from the slides using 70% ethanol followed by 100% ethanol; both steps were conducted for 2 min each. The slides that had been used for DESI did not require this matrix removal step. Both MALDI and DESI slides were then stained with hematoxylin and eosin (H&E). Optical images of these slides were acquired using a 20× objective magnification on a Zeiss Axioscan 7 (Zeiss, Germany).

### Data Processing

The DESI acquired images were processed using high-definition imaging (HDI) software (Waters Corporation, UK) for initial observations of the data. For full analysis and comparison against the proteomic target list, the DESI and MALDI files were moved into SCiLS Lab (Bruker, USA). The total ion count (TIC) normalization method was used to scale the intensity for the images. Following the analysis against the proteomic target list and extraction of the resulting images from SCiLS Lab (Bruker, USA), the DESI spectra were transferred back to high-definition imaging (HDI) software (Waters Corporation, UK) to allow visualization of the ion mobility separated spectra. The mobiligram was then exported from HDI (Waters Corporation, UK) to DriftScope (Waters Corporation, UK), where the spectra were further processed. The DESI spectra were then exported to Mass Lynx (Waters Corporation, UK) retaining the drift time. Finally, both the DESI and MALDI spectra were copied into GraphPad Prism (GraphPad Software, USA) to allow comparison of the techniques.

The rat peptide target list was generated using Genedata Expressionist (Genedata, Switzerland), whereas the mouse peptide target list was generated using Proteome Discoverer version 2.5 (Thermo Scientific, USA). The MS/MS data was searched against a Swiss-Prot (Swiss Institute of Bioinformatics, Switzerland) proteome database for *Rattus norvegicus* and *Mus musculus*, depending on whether the rat or mouse brain was used. The cerebellum and the rest of the brain target lists for the respective mouse and rat brains were combined into one list. These target lists were then imported into SCiLS Lab (Bruker, USA) to filter against the possible peptide images acquired from the mass spectrometry imaging experiments. Additionally, any cysteine-containing peptides were removed from the target list to adjust for carbamidomethylation. This gave a list of on-tissue tryptic peptides that matched the *m*/*z* on the target list. Thus, the obtained peptides were used to infer corresponding proteins.

## Results and Discussion

**Figure 1:**
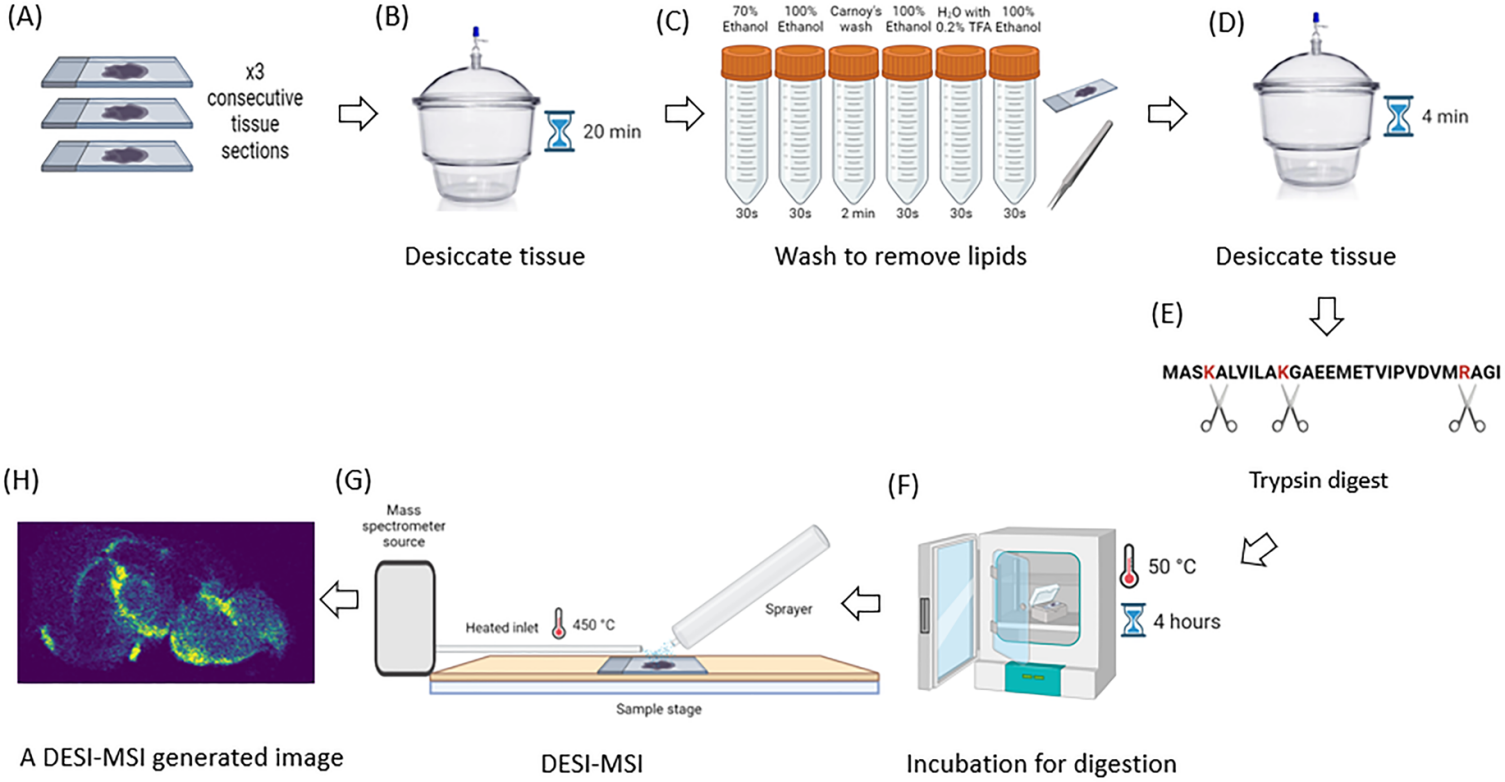
Schematic of the DESI-MSI method for generating images of spatially localized tryptic peptides from tissue. (A) Consecutive tissue sections with alternate sections for DESI-MSI and MALDI-MSI. For both techniques, ×3 replicates were used for each; this was done for both mouse and rat brain sections. (B) Desiccation of tissue sections to prevent peptide/protein delocalization by condensation. (C) Washing the tissue using Carnoy’s wash to remove interfering ions such as lipids. (D) Desiccation of tissue sections to remove residual wash solution. (E) Trypsin was applied to digest the proteins into peptides. (F) Incubated to allow the digestion of the proteins in the tissue. (G) Oriented the sprayer and sample stage of the DESI for maximum peptide ion retrieval into the mass spectrometer. (H) An example of a resulting DESI-MSI tryptic peptide image at 50 μm resolution. Created with BioRender.com.

We sought to demonstrate that localized tryptic peptides can be detected, and the resulting images have a higher spatial resolution than shown previously with DESI-MSI. To achieve this, the mouse and rat brain tissue from consecutive sections was assigned to alternate DESI-MSI and MALDI-MSI experiments. This was done for ×3 replicates of each technique and for both tissue types (Figure 1A). These tissue sections were desiccated for 20 min after freezer removal to prevent peptide and protein delocalization through condensation (Figure 1B). Next, the sections were washed using a combination of Carnoy’s wash solution and ethanol to remove interfering ions such as lipids and salts (Figure 1C). Vacuum desiccation was used for 4 min to remove the remaining wash solution and prevent delocalization of the peptides and proteins (Figure 1D). Trypsin was then applied to the tissue to generate the peptides (Figure 1E). Once the trypsin had been added the tissue sections were moved to a 50 °C incubation for 4 h (Figure 1F). Next, the sections were transferred to the DESI source with a heated inlet at approximately 450 °C, used to optimize the generation of multiply charged analyte ions (Figure 1G). This method resulted in a high number of localized tryptic peptide images at multiple charge states (Figure 1H). The notable differences compared to some previous methods include: the desiccation time, trypsin incubation time,^42^ the optimization of ion mobility settings, and the orientation of the components in the DESI source.^21^ All these steps have previously been introduced by other groups but were further optimized for this protocol. Here, an optimized method is presented that allows frozen tissue sections to be used for the detection of highly localized tryptic peptides using DESI-MSI.

### Precise Localization of Tryptic Peptides in Tissue with High Spatial Resolution by DESI-MSI

**Figure 2:**
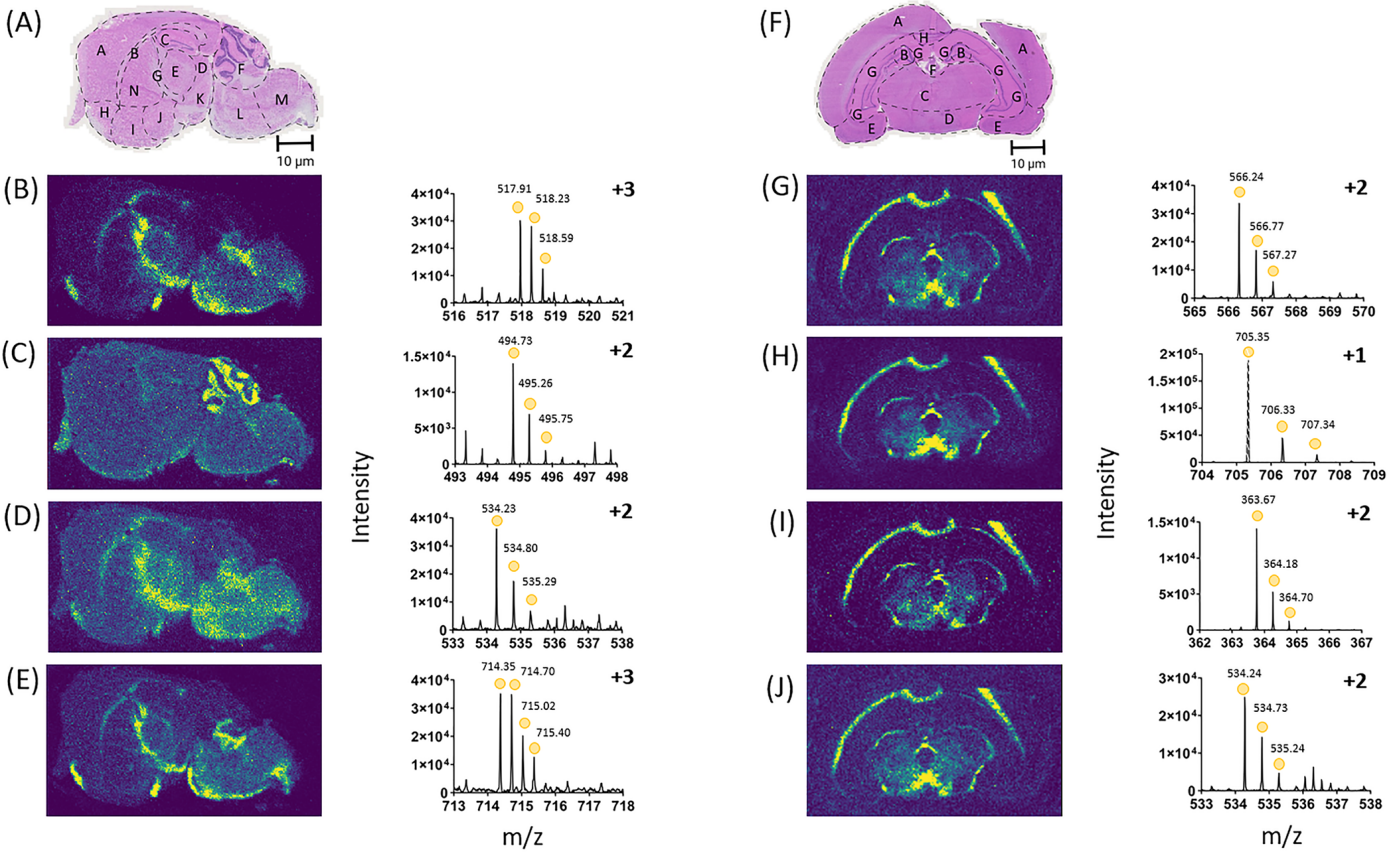
Examples of DESI-MSI generating images that show the precise localization of tryptic peptides from mouse and rat brain sections at 50 μm. These were acquired using a heated inlet and ion mobility. (A) H&E stain of a consecutive section of mouse brain after DESI-MSI, with regions of the brain highlighted. For the mouse brain, A - cerebral cortex, B - corpus callosum, C - hippocampus, D - midbrain, E - thalamus, F - cerebellum, G - fornix, H - anterior olfactory nucleus, I - ventral striatum, J - basal forebrain, K - hypothalamus, L - pons, M - medulla, N - caudate putamen. For the mouse brain sections the tryptic peptides detected are included. (B) ATP synthase subunit alpha (Peptide ID EAYPGDVFYLHSR), 517.9128 *m*/*z* ± 54.9 ppm. (C) Pyruvate dehydrogenase E1 component subunit alpha (Peptide ID - AAASTDYYK), 494.7287 *m*/*z* ± 84.2 ppm. (D) Tubulin beta-3 chain, (Peptide ID - YLTVATVFR), 534.8020 *m*/*z* ± 57.3 ppm. (E) V-type proton ATPase catalytic subunit A, (Peptide ID - ALDEYYDKHFTEFVPLR), 714.3498 *m*/*z* ± 31.3 ppm. This was shown in another tissue type of rat brain, where DESI-MSI generated images that show the precise localization of tryptic peptides at 100 μm. (F) H&E stain of a consecutive section of rat brain after DESI-MSI, with regions of the brain highlighted. For the rat brain, A - neocortex, B - hippocampal dentate gyrus, C - thalamus, D - hypothalamus, E - amygdaloid nucleus, F - habenular nucleus, G - cornu ammonis, H - corpus collosum. (G) 14-3-3 protein epsilon (Peptide ID - YDEMVESMK), 566.2386 *m*/*z* ± 48.3 ppm. (H) Regulating synaptic membrane exocytosis protein 2 (Peptide ID - QMGVSGK), 705.3462 *m*/*z* ± 13.7 ppm. (I) Secretory carrier-associated membrane protein 5 (Peptide ID - GSGGSFSK), 363.6736 *m*/*z* ± 50.1 ppm. (J) Homer protein homologue 1 (Peptide ID - SQSEQDAFR), 534.2390 *m*/*z* ± 64.0 ppm.

We sought to demonstrate that DESI-MSI could be used to detect highly localized tryptic peptides in multiple tissue types. The distribution of tryptic peptides detected in these consecutive tissue sections for DESI-MSI was found to be highly localized in specific regions of tissue, with an image spatial resolution of 50 μm (Figure 2). The identified peptides were then searched against a target list derived from the same tissue that had been homogenized and subjected to proteomic analysis. The corresponding proteins assigned from this were then correlated where possible to literature. Corresponding proteins and tryptic peptides detected in these images for the mouse brain sections included (Figure 2): pyruvate dehydrogenase E1 component subunit alpha (Peptide ID - AAASTDYYK, Uniprot - P35486) with this form of the tryptic peptide located exclusively in the cerebellum (Figure 2C). ATP synthase subunit alpha (Figure 2B) (Peptide ID - EAYPGDVFYLHSR, Uniprot - Q03265), tubulin beta-3 chain (Figure 2D) (Peptide ID - YLTVATVFR, Uniprot - Q9ERD7) and v-type proton ATPase catalytic subunit A (Figure 2E), (Peptide ID - ALDEYYDKHFTEFVPLR, Uniprot - P50516) were detected through the corpus callosum down to the medulla. Conversely, tubulin beta-3 chain (Figure 2) seems to be more concentrated in the midbrain than the ATP synthase subunit alpha and v-type proton ATPase catalytic subunit A. The peptides identified were all found in regions supported by the literature.^43−46^

**Figure 3:**
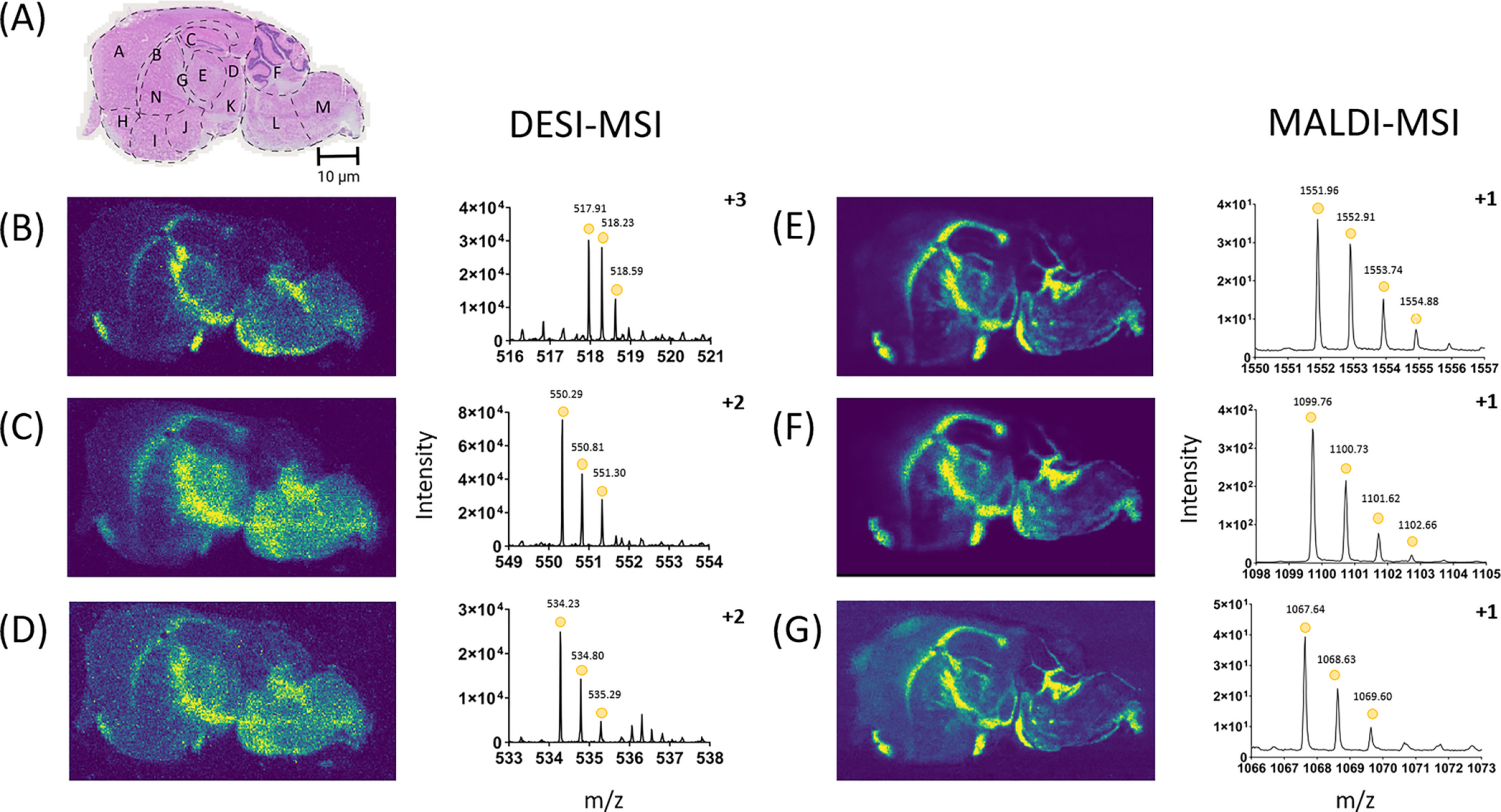
DESI-MSI is comparable to MALDI-MSI for the detection of some peptides, as similar localization is observed for these example peptides in mouse brain sections at 50 μm. Therefore, DESI-MSI can be used alongside MALDI-MSI for peptide confirmation. (A) H&E stain of a consecutive section of mouse brain after DESI-MSI, with regions of the brain highlighted. For the mouse brain, A - cerebral cortex, B - corpus callosum, C - hippocampus, D - midbrain, E - thalamus, F - cerebellum, G - fornix, H - anterior olfactory nucleus, I - ventral striatum, J - basal forebrain, K - hypothalamus, L - pons, M - medulla, N - caudate putamen. (B) ATP synthase subunit alpha (Peptide ID - EAYPGDVFYLHSR), 517.9128 *m*/*z* ± 54.9 ppm. (C) Spectrin alpha chain, nonerythrocytic 1 (Peptide ID - DLAALEDKVK), 550.8075 *m*/*z* ± 72.8 ppm. (D) Tubulin beta-3 chain, (Peptide ID - YLTVATVFR), 534.8020 *m*/*z* ± 57.3 ppm. (E) ATP synthase subunit alpha (Peptide ID - EAYPGDVFYLHSR), 1553.7380 *m*/*z* ± 32.4 ppm. (F) Spectrin alpha chain, nonerythrocytic 1 (Peptide ID - DLAALEDKVK), 1101.6150 *m*/*z* ± 58.0 ppm. (G) Tubulin beta-3 chain, (Peptide ID - YLTVATVFR), 1069.6041 *m*/*z* ± 36.8 ppm.

**Figure 4:**
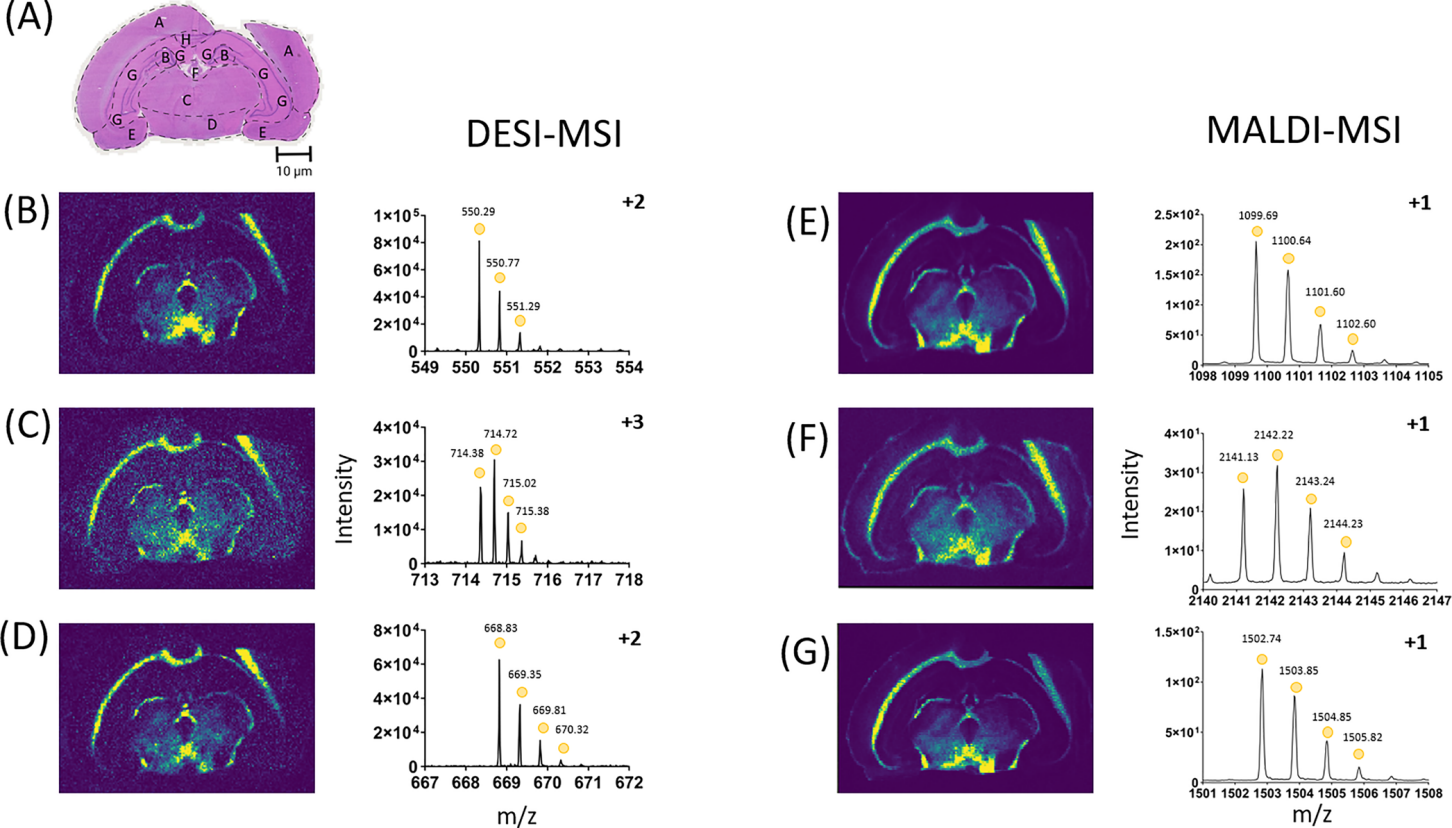
DESI-MSI is comparable to MALDI-MSI for the detection of some peptides as similar localization is observed for these example peptides in rat brain sections at 100 μm. Therefore, DESI-MSI can be used alongside MALDI-MSI for peptide confirmation. (A) H&E stain of a consecutive section of rat brain after DESI-MSI. with regions of the brain highlighted. For the rat brain, A - neocortex, B - hippocampal dentate gyrus, C - thalamus, D - hypothalamus, E - amygdaloid nucleus, F - habenular nucleus, G - cornu ammonis, H - corpus collosum. (B) Gamma-enolase (Peptide ID - DGKYDLDFK), 550.7667 *m*/*z* ± 79.6 ppm. (C) Proteasome subunit beta type-3 (Peptide ID - QIKPYTLMSMVANLLYEK), 714.7157 *m*/*z* ± 51.9 ppm. (D) Clathrin heavy chain 1, (Peptide ID - VVGAMQLYSVDR), 669.3466 *m*/*z* ± 33.2 ppm. (E) Gamma-enolase (Peptide - DGKYDLDFK), 1099.5190 *m*/*z* ± 98.0 ppm. (F) Proteasome subunit beta type-3 (Peptide ID - QIKPYTLMSMVANLLYEK), 2141.1250 *m*/*z* ± 103.2 ppm. (G) Clathrin heavy chain 1, (Peptide ID - MREHLELFWSR), 1502.7444 *m*/*z* ± 99.1 ppm.

**Figure 5:**
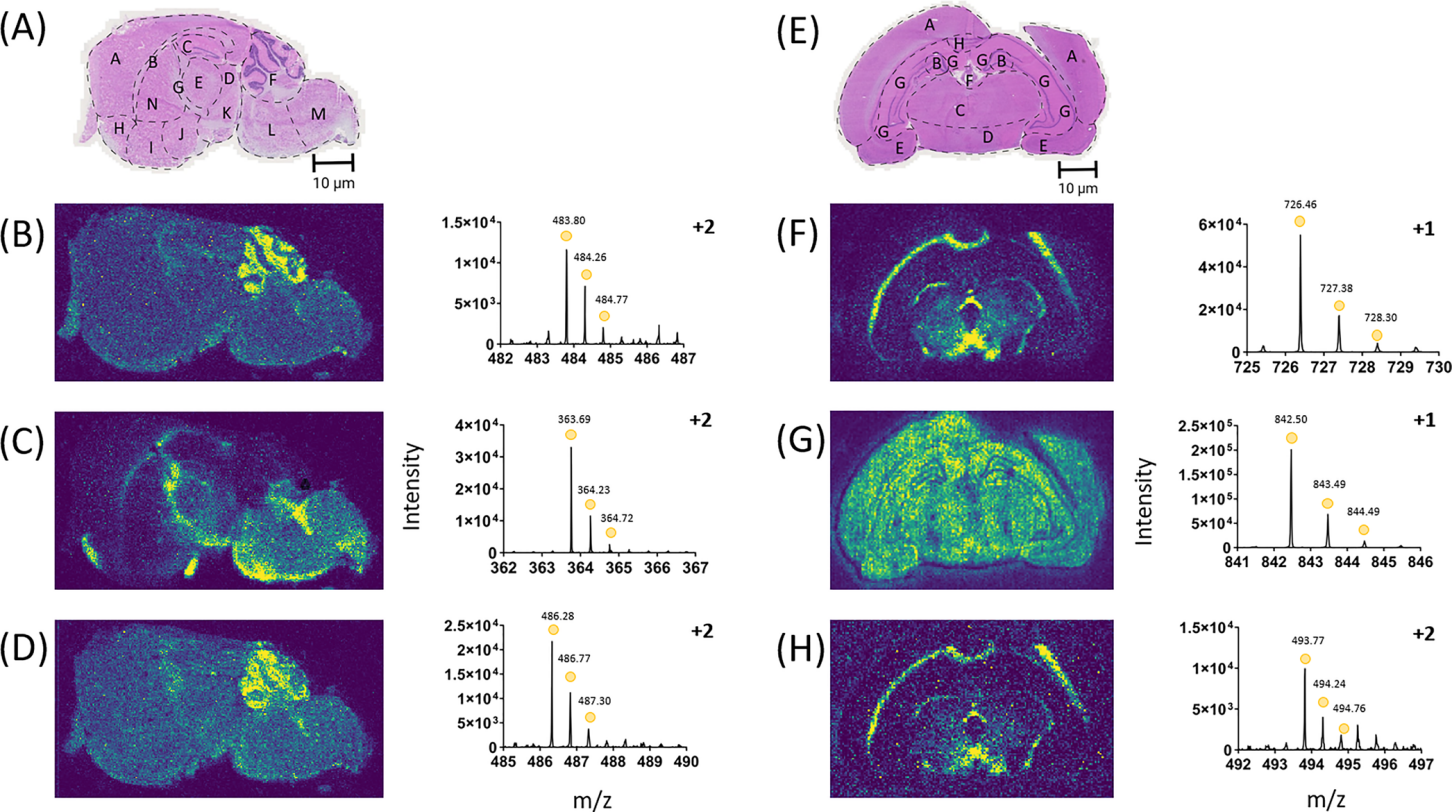
DESI-MSI can identify peptides that have not been detected using MALDI-MSI in the mouse and rat brain for these replicates used at 50 μm. This shows the benefit of using DESI-MSI to detect lower mass multiply charged peptides, showing that the technique can be complementary to MALDI-MSI. (A) H&E stain of a consecutive section of mouse brain after DESI-MSI, with regions of the brain highlighted. For the mouse brain, A - cerebral cortex, B - corpus callosum, C - hippocampus, D - midbrain, E - thalamus, F - cerebellum, G - fornix, H - anterior olfactory nucleus, I - ventral striatum, J - basal forebrain, K - hypothalamus, L - pons, M - medulla, N - caudate putamen. For the mouse brain sections the tryptic peptides detected included, (B) Rap1 GTPase-GDP dissociation stimulator 1 (Peptide ID - NLAIPVVNK), 483.7967 *m*/*z* ± 44.0 ppm. (C) Plasma membrane calcium-transporting ATPase 4, (Peptide ID - LAVQIGK), 364.2333 *m*/*z* ± 34.0 ppm. (D) Arf-GAP with SH3 domain, ANK repeat and PH domain-containing protein 2, (Peptide ID - EIISEVQR), 486.7656 ± 33.9 ppm. DESI-MSI can identify peptides that have not been detected using MALDI-MSI in another tissue, the rat brain for these replicates used at 100 μm. This demonstrates the co-operativity of DESI with MALDI in identifying the corresponding proteins for the peptides in the tissue. (E) H&E stain of a consecutive section of rat brain after DESI-MSI, with regions of the brain highlighted. For the rat brain, A - neocortex, B - h dentate gyrus, C - t, D - hypothalamus, E - amygdaloid nucleus, F - habenular nucleus, G - cornu ammonis, H - corpus collosum. (F) Ubiquitin-conjugating enzyme E2 variant 2 (Peptide ID - SIPVLAK), 726.4625 *m*/*z* ± 96.8 ppm. (G) Ras/Rap GTPase-activating protein SynGAP (Peptide ID - RVDNVLK), 842.4964 *m*/*z* ± 98.3 ppm. (H) Protein disulfide-isomerase (Peptide ID - QLAPIWDK), 493.7663 *m*/*z* ± 20.0 ppm.

**Figure 6:**
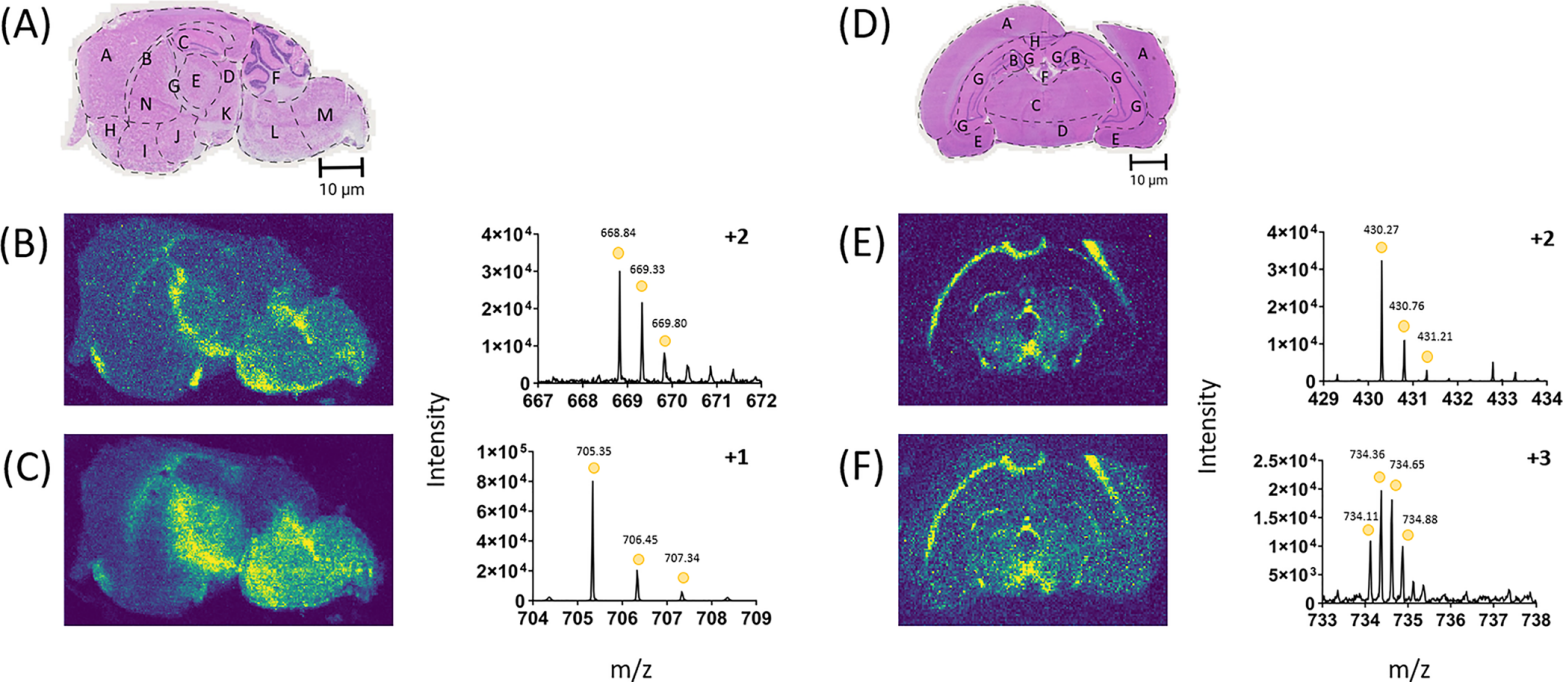
DESI-MSI can identify multiple tryptic peptides that correspond to the same protein in both the mouse at 50 μm and rat brain tissue. These images are examples of this, improving confidence in the protein ID’s. (A) H&E stain of a consecutive section of mouse brain after DESI MSI, with regions of the brain highlighted. For the mouse brain, A - cerebral cortex, B - corpus callosum, C - hippocampus, D - midbrain, E - thalamus, F - cerebellum, G - fornix, H - anterior olfactory nucleus, I - ventral striatum, J - basal forebrain, K - hypothalamus, L - pons, M - medulla, N - caudate putamen. Tryptic peptides identified for the mouse brain were (B) Clathrin heavy chain 1, (Peptide ID - VVGAMQLYSVDR), 668.8441 ± 53.1 ppm (C) Clathrin heavy chain 1, (Peptide ID - IVLYAK), 706.4498 ± 59.5 ppm. (D) H&E stain of a consecutive section of rat brain after DESI MSI, with regions of the brain highlighted at 100 μm. For the rat brain, A - neocortex, B - hippocampal dentate gyrus, C - thalamus, D - hypothalamus, E - amygdaloid nucleus, F - habenular nucleus, G - cornu ammonis, H - corpus collosum. Tryptic peptides identified that correspond to the same protein for the rat brain are (E) AP-2 complex subunit beta, (Peptide ID - FLELLPK), 430.2681 *m*/*z* ± 59.4 ppm and (F) AP-2 complex subunit beta, (Peptide ID - LHDINAQMVEDQGFLDSLR), 734.3595 *m*/*z* ± 46.7 ppm.

Representative DESI-MS mass spectra for the specific images selected are also shown (Figures 2–6). For all the DESI-MSI, ion mobility was applied to resolve the peaks. Therefore, the images and spectra shown are binned for ion mobility. Ion mobility binning was conducted in HDI (Figures 2–6), encompassing drift times ranging from 40 to 140 bins. These images and spectra (Figure 2) show that DESI-MSI can generate images of highly localized tryptic peptides in different regions of the tissue.

The optimized DESI-MSI protocol was then applied on rat brain sections to see if multiple, highly localized peptides could be detected from an additional tissue source (Figure 2). The images acquired had a pixel size of 100 μm, which was a lower resolution than the mouse brain but still higher than previous work that obtained a pixel size of 300 μm^33^ and 150 μm.^21^ The pixel size was changed due to the increased tissue area, which increased acquisition time. The image quality was still comparable to the 50 μm images acquired with the mouse brain. Putatively assigned tryptic peptides of 14-3-3 protein epsilon (Figure 2G) (Peptide ID - YDEMVESMK, Uniprot - P62260), regulating synaptic membrane exocytosis protein 2 (Figure 2H) (Peptide ID - QMGVSGK, Uniprot - Q9JIS1), (Figure 2I) secretory carrier-associated membrane protein 5 (Peptide ID - GSGGSFSK, Uniprot - Q9JKE3), and (Figure 2J) homer protein homologue 1 (Peptide ID - SQSEQDAFR, Uniprot - Q9Z214) were all present in the same orientation in the brain. These peptides were localized to the thalamus and hypothalamus. The location of 14-3-3 protein epsilon and secretory carrier-associated membrane protein 5 was confirmed in literature.^47,48^ A clear margin can be observed around the tissue for both mouse and rat brain images, indicating that delocalization has not occurred as the identified tryptic peptides are only found on the tissue, not on the surrounding slide. This demonstrated that in another tissue type, highly localized tryptic peptides could be detected using DESI-MSI.

### Potential for Increased Numbers of Tryptic Peptide Ions Detected by DESI-MSI Compared to MALDI-MSI

Until recently, DESI-MSI has only resulted in the detection of a small number of specifically localized tryptic peptides in tissue.^21^ A protocol for identifying large numbers of localized tryptic peptides in tissue using DESI-MSI has not yet been developed. Due to this, DESI-MSI was optimized for this study to gain a high number of localized potential tryptic peptide ions. Detected ions were filtered against the proteomic target lists, generated by LC–MS (Thermo Exploris 240), allowing tentative protein assignment for each peptide ion image. This list contained the proteins that corresponded to the peptide ions detected in a proteomics experiment on the same rat and mouse brain tissue used in the imaging experiments. It was filtered against both the MALDI and DESI imaging experiments to provide corresponding protein names for the peptide ions identified. The numerical data set was filtered and segmented in SCiLS initially; however, this still contained some multiple image assignments for each mass identified and other images showing only partial segmentation on the tissue. Therefore, additional filtering was conducted whereby multiple peptide ions corresponding to the same image and any peptide ions showing any off-tissue signal were discarded.

Overall, this filtering allowed a more accurate comparison of the possible localized tryptic peptide ions from both techniques (Table S1). Filtering effectiveness has been demonstrated, as the number of peptide ions drastically decreases once this filtering is applied, with any dubiously assigned peptide ions removed. Possible peptide ion numbers are reduced by 80% for the MALDI-MSI mouse brain tissue, 59% for the DESI-MSI mouse brain tissue, 97% for the MALDI-MSI rat brain tissue, and 76% for the DESI-MSI rat brain tissue (Table S1). This shows the benefit of manually removing possible peptide ions that show multiple image assignments per mass and poor segmentation.

Large numbers of potential localized tryptic peptide ions were obtained for both tissue types in DESI-MSI and MALDI-MSI (Table 1). The filtered data shows that DESI-MSI leads to a greater number of localized, non-overlapping peptide ions than MALDI-MSI in both tissue types, primarily due to multiple charge state detection in DESI-MSI, in comparison to MALDI-MSI that only generates singly charged species. For the mouse brain sections, MALDI-MSI resulted in only 21% of the number of potential tryptic peptide ions identified with DESI-MSI, whereas for the corresponding rat brain sections this was only 7%. As DESI-MSI can detect multiply charged species, it can also detect the lower mass to charge peptides that MALDI-MSI is unable to detect, leading to a greater number of peptide ions per protein identified. The difference between mouse and rat brain numbers is partially due to tissue freshness which seemed to be more important for DESI-MSI rather than MALDI-MSI. This pertains to the duration for which the tissue has been stored in the −80 °C freezer post sacrifice. As the storage period increases, there is a decrease in the number of detected tryptic peptides. Tissue quality was a factor of greater importance for tryptic peptide detection rather than lipid detection, as spatially localized lipid detection is usually successful with DESI-MSI regardless of tissue quality. The significance of tissue freshness in the detection of tryptic peptides has not been reported previously.

**Table 1:** Numerical Comparison of Tentative Tryptic Peptide Ions Detected Using DESI-MSI when Compared to MALDI-MSI for These Replicates^a^

Type of experiment	Number of potential tryptic peptide ions detected
MALDI-MSI—Mouse brain tissue	709
DESI-MSI—Mouse brain tissue	3,367
MALDI-MSI—Rat brain tissue	263
DESI-MSI—Rat brain tissue	3,591

a The table shows post manual filtering of these ions in SCiLS against a proteomic target list. This filtering only retains those fully localized on the tissue with no multiple assignments for the *m*/*z* in the target list. The corresponding proteins for these peptides were identified in Uniprot using an LC–MS proteomic run of successive tissue sections. These numbers are from the replicates used in the experiments with consistent conditions between these. All the peptide assignments included in the table have not been individually validated; however, the initial mascot search based on the proteomic target list indicates these are correct.

These numbers are specifically for the conditions used in these experiments and may vary depending on DESI set up and tissue quality. This difference could also be partially due to ion mobility being applied to DESI-MSI and not MALDI-MSI, which could result in a greater number of peptides being identified. These differences were observed based on the instrument configuration available for this study; if ion mobility was combined with MALDI-MSI,^49,50^ the interfering matrix adduct ions would be reduced, potentially resulting in greater peptide ion detection. Overall, for the detection of potential tryptic peptide ions, DESI-MSI detects a greater number than for MALDI-MSI.

### Direct Comparison of Localized Tryptic Peptides from Tissue Sections by DESI-MSI and MALDI-MSI

The aim of this work was to compare tryptic peptides found in MALDI-MSI to those found in DESI-MSI and see if the peptide localizations from the two techniques correlate. The localization of peptides detected using DESI-MSI has not been comparable to the high-resolution images obtained using MALDI-MSI in previous work.^51,52^ Images showing comparable spatial localization of an ion at the same image resolution in both imaging techniques would allow greater confidence in tryptic peptide and subsequent protein identification. For this reason, we compared images of tryptic peptides found in both the mouse and rat brain sections for DESI-MSI to the same peptides found in MALDI-MSI from alternate tissue sections.

Three tryptic peptides were selected to demonstrate precise localization in mouse brain sections for DESI-MSI that were of comparable quality to those generated using MALDI-MSI to allow a direct comparison between the techniques (Figure 3). ATP synthase subunit alpha (Figure 3B,E) (Peptide ID - EAYPGDVFYLHSR, Uniprot - Q03265), spectrin alpha chain nonerythrocytic 1 (Figure 3C,F) (Peptide ID - DLAALEDKVK, Uniprot - P16546), and tubulin beta-3 chain (Figure 3D,G), (Peptide ID - YLTVATVFR, Uniprot - Q9ERD7) were localized in both techniques through the corpus callosum down to the medulla as observed in the literature.^43,45^ Overall, the ion images show a very similar localization (Figure 3), which would allow DESI-MSI to be used alongside MALDI-MSI, increasing the number of peptides detected, and to further improve confidence in the MALDI-MSI detection.

To demonstrate this in another tissue, the rat brain sections with discrete distribution profiles were compared between DESI-MSI and MALDI-MSI (Figure 4). The same tryptic peptides showing corresponding localization in both techniques were gamma-enolase (Figure 4B,E) (Peptide ID - DGKYDLDFK, Uniprot - P07323) and proteasome subunit beta type-3 (Figure 4C,F) (Peptide ID - QIKPYTLMSMVANLLYEK, Uniprot - P40112). Some peptides identified in the same orientation for both DESI-MSI and MALDI-MSI were different tryptic peptide sequences that corresponded to the same protein. Clathrin heavy chain 1 is an example in which the peptides identified were different, but both corresponded to the same protein (Figure 4D,G). Therefore, one peptide had an ID of VVGAMQLYSVDR, but the other had an ID of MREHLELFWSR with both having the same corresponding Uniprot ID of P11442. The distribution for all these corroborating peptides is through the corpus collosum, thalamus, and hypothalamus, with notable peptide absence from the central septal nuclei. This corresponds to previous literature for gamma-enolase and clathrin heavy chain 1, both found in the hypothalamus.^53,54^ This demonstrates that DESI-MSI can be used to confirm tryptic peptides found in MALDI-MSI.

### Tryptic Peptides with High Spatial Localization Only Found in DESI-MSI That Were Not Detected in a Localized Form in MALDI-MSI

Tryptic peptides are commonly detected using MALDI-MSI, however, generally only as singly charged species, so this can reduce the total number detected when compared to DESI-MSI that can detect multiply charged species. Hence, the detection of multiply charged species in DESI-MSI increases the likelihood of identifying the species of interest, as it allows for the detection of multiple charge states of a peptide across a defined mass range. DESI-MSI has not previously been shown to detect specific localized peptides that are not detected in MALDI-MSI. We investigated whether DESI-MSI can identify tryptic peptides that are not always found in MALDI-MSI. These ions were compared against the proteomic target list and were found to be discretely localized within the tissues analyzed.

Examples of tryptic peptides detected for the mouse brain sections in DESI-MSI but not in MALDI-MSI (Figure 5) were assigned rap1 GTPase-GDP dissociation stimulator 1 (Figure 5B) (Peptide ID - NLAIPVVNK, Uniprot - E9Q912), plasma membrane calcium-transporting ATPase 4 (Figure 5C) (Peptide ID - LAVQIGK, Uniprot - Q6Q477), and arf-GAP with SH3 domain, ANK repeat and PH domain-containing protein 2 (Figure 5D) (Peptide ID - EIISEVQR, Uniprot - Q7SIG6). Rap1 GTPase-GDP dissociation stimulator 1 was localized to the cerebellum only, with no signal coming from anywhere else in the tissue. Arf-GAP with SH3 domain, ANK repeat, and PH domain-containing protein 2 was localized to the cerebellum with slight delocalization from this area.^55^ Plasma membrane calcium-transporting ATPase 4 was localized throughout the corpus collosum, along the internal capsule down to the pons and medulla. For Arf-GAP and plasma membrane calcium-transporting ATPase 4, the peptides identified were found in regions supported by literature.^56,57^ Here, we determined that DESI-MSI was able to detect certain tryptic peptide ions not found in the MALDI-MSI data sets.

The same analysis for ion image examples from the rat brain tissue was conducted, also showing certain tryptic peptides were detected using DESI-MSI but not MALDI-MSI (Figure 5). These were tentatively assigned to the proteins, ubiquitin-conjugating enzyme E2 variant 2 (Figure 5F) (Peptide ID - SIPVLAK, Uniprot - Q7M767), ras/rap GTPase-activating protein SynGAP (Figure 5G) (Peptide ID - RVDNVLK, Uniprot - Q9QUH6), and protein disulfide-isomerase (Figure 5H) (Peptide ID - QLAPIWDK, Uniprot - P04785). Ubiquitin-conjugating enzyme E2 variant 2 and protein disulfide-isomerase were both localized to the corpus collosum, thalamus, and the hypothalamus, with notable peptide absence from the central septal nuclei. Ras/Rap GTPase-activating protein SynGAP was not particularly localized in a specific area but showed absence from the border around the hippocampal dentate gyrus. The peptide location of ras/rap GTPase-activating protein SynGAP was confirmed in literature.^58^ Detection of peptides in DESI-MSI but not MALDI-MSI could be due to ion mobility being utilized in DESI-MSI and not MALDI-MSI, as an additional level of separation and deconvolution of data was possible. Ion mobility is not indispensable for the analysis of proteins and peptides through DESI-MSI. Nevertheless, if the goal is to differentiate overlapping charge states and ensure confidence in the identification of proteins and peptides, then ion mobility becomes necessary.

These examples of peptides found in both mouse and rat brain sections for DESI-MSI all have lower than 900 *m*/*z* and are mostly multiply charged species, easily detected in DESI-MSI. These peptides would most likely not be detected using MALDI-MSI, unless the singly charged species of this peptide was also generated. Further, the ionization process in MALDI-MSI generates matrix cluster ions and fragments that are usually at a lower molecular weight of <600 *m*/*z*, interfering with any lower molecular weight ions from the tissue.^31^ This demonstrates a potential application for DESI-MSI being utilized alongside MALDI-MSI to detect smaller mass peptides and larger multiply charged peptides.

### Multiple Unique Tryptic Peptides of the Same Corresponding Protein That Demonstrate Similar Localization

Tryptic peptide identification for specific peptides of interest will require further steps to increase confidence in the ID’s. The aim of this work was to improve the validity of the preliminary assignment of the tryptic peptides. To bolster the tentative protein ID’s assigned, some example images of multiple corroborating tryptic peptides that demonstrate the same localization for their inferred protein are shown (Figure 6). For the mouse brain sections, one of the examples identified was clathrin heavy chain 1 (Uniprot - Q68FD5) (Figure 6). The tryptic peptides that matched this protein, both showed the same localization throughout the corpus collosum through the internal capsule down to the pons and medulla, corresponding to previous work.^53^ These peptides corresponding to clathrin heavy chain 1 were VVGAMQLYSVDR (Figure 6B) and IVLYAK (Figure 6C). This shows that the corresponding protein of clathrin heavy chain 1 is likely to be found in this localization, as it has multiple peptides confirming this.

This corroborative identification with multiple peptides of the same protein was also observed in the other tissue type of rat brain (Figure 6); one example is AP-2 complex subunit beta. The tryptic peptides corresponding to this protein were FLELLPK (Figure 6E) and LHDINAQMVEDQGFLDSLR (Figure 6F). The peptides were found in the corpus collosum, the thalamus, and the hypothalamus, with signal absence from the central septal nuclei as identified in the literature.^53^ For both mouse and rat brain identified peptides, the localization is slightly different in the images. This is likely due to the differing intensity between the two comparisons; therefore, the signal is not high enough in some regions of the image to show localization. Illustrating example images of a couple of unique peptides that correspond to the same protein with similar localization patterns, increases confidence in the assignment. This should be conducted where possible to improve the reliability of the tentative protein ID’s. This coupled with the optimizations discussed above, leads to well resolved images of tryptic peptides detected using DESI-MSI, that can be tentatively assigned to protein IDs.

## Conclusions

Improved detection of tryptic peptides in tissue using DESI-MSI at higher spatial resolution than shown previously has been achieved. The spatial resolution of these images has been enhanced, with a resolution of 50 μm for the mouse brain and 100 μm for the rat brain, an improvement from the current documented resolution of 150 μm. This optimized DESI-MSI workflow has led to considerable improvement in the number of tryptic peptides that can be detected from both mouse and rat brain tissues analyzed. This DESI-MSI workflow resulted in a 4.75-fold increase in potential tryptic peptide ions for mouse brain sections and a 13.66-fold increase for rat brain sections when compared to MALDI-MSI. Specifically, in this study we detected 3,367 and 3,591 potential tryptic peptide ions with DESI-MSI, in contrast to MALDI-MSI that detected 709 and 263 for the mouse and rat brain tissue sections, respectively. Naturally, these numbers are anticipated to be subject to significant variability, as they are specific to the conditions used in these experiments. Future studies may detect varying numbers of potential tryptic peptide ions based on tissue type, tissue quality, DESI setup, and various other experimental factors.

The use of an LC–MS derived proteomic target list allowed these spatially resolved tryptic peptide ions to be tentatively identified. DESI-MSI can be used alongside MALDI-MSI for tryptic peptide confirmation, allowing drug targets to be detected in the tissue with greater certainty.

DESI-MSI shows the potential for detecting tryptic peptide species that are multiply charged and as such not routinely found in MALDI-MSI, increasing the scope for overall detection when combining the two modalities. Multiple unique peptide images showing similar localization patterns for the same corresponding protein have been identified, further improving the reliability of the protein IDs.

DESI-MSI could be enhanced to improve the image resolution, allowing additional species that are only present in smaller, highly localized regions of the brain to be detected. To validate the differences between the numbers of tryptic peptides detected between DESI-MSI and MALDI-MSI, the same instrument should be used for both with ion mobility capabilities. This would allow determination of how great an effect ion mobility has on the number of tryptic peptides and how much is due to DESI-MSI generating multiply charged peptides.

## Supporting Information Available

Table with the numbers of tentative tryptic peptide ions detected before and after manual filtering using DESI-MSI when compared to MALDI-MSI for these replicates. Figure showing the DESI-MSI source (Prosolia, USA) setup (PDF)
