# Supplementary material for: Improved Detection of Tryptic Peptides from Tissue Sections Using Desorption Electrospray Ionization Mass Spectrometry Imaging
Source: J Am Soc Mass Spectrom. 2024 Apr 11;35(5):922–34. doi: 10.1021/jasms.4c00006 (PMC11066963; doi:10.1021/jasms.4c00006)
Supplement: Supplementary file 1 — js4c00006_si_001.pdf [file js4c00006_si_001.pdf]

Supporting Information

Improved detection of tryptic peptides from tissue sections using Desorption electrospray ionisation mass spectrometry imaging (DESI-MSI)

Heather Bottomley[1], Jonathan Phillips[1], Philippa Hart[2]*

[1]Living Systems Institute, Department of Biosciences, University of Exeter, Stocker Road, Exeter, EX4 4QD.

[2]Medicines Discovery Catapult, Alderley Park, Block 35, Mereside, Macclesfield, SK10 4ZF

*Corresponding author: Philippa.hart@md.catapult.org.uk

Suppl. Table 1: Table with the numbers of tentative tryptic peptide ions detected before and after manual filtering using DESI-MSI when compared to MALDI-MSI for these replicates. The corresponding proteins were identified in Uniprot using a LC-MS proteomic run of successive tissue sections. These numbers are from the replicates used in the experiments with consistent conditions between these. All the tryptic peptide ion assignments included in the table have not been individually validated, however the initial mascot search based on the proteomic target list indicates these are correct.

Type of experiment	Number of potential tryptic peptide ions before manual filtering	Number of potential tryptic peptide ions after manual filtering
MALDI-MSI - Mouse brain tissue	3,487	709
DESI-MSI – Mouse brain tissue	8,283	3,367
MALDI-MSI – Rat brain tissue	10,042	263
DESI-MSI – Rat brain tissue	15,224	3,591

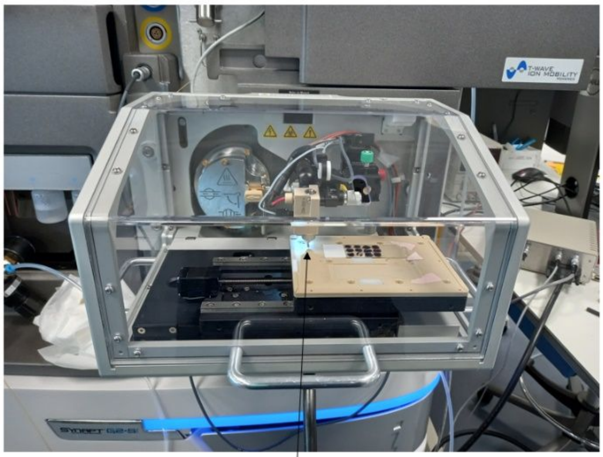

Pre-commercial heated inlet
(approx. 450 °C) to the mass
spectrometer.

Suppl. Figure 1: DESI-MSI source (Prosolia, USA) consisting of a moving sample stage, sprayer nozzle and a pre-commercial heated inlet (approx. 450 °C) to the mass spectrometer.
